# Texting for health in the safety net: Improving health promotion and outreach

**DOI:** 10.1186/1748-5908-10-S1-A82

**Published:** 2015-08-20

**Authors:** Susan L Moore, Henry H Fischer, Arthur J Davidson, Julia Dorado-Cole, Forrest D Pugh, Susan Becker, Curt Straub, Jeffrey D Berschling, Tracy Johnson

**Affiliations:** 1 Denver Health and Hospital Authority, Denver, CO 80204, USA; 2 Department of Medicine, University of Colorado Denver School of Medicine, University of Colorado Denver, Aurora, CO 80045, USA; 3 Colorado School of Public Health, University of Colorado Denver, Aurora, CO 80045, USA; 4 EMC2 Consulting, EMC Corporation, Hopkinton, MA 01748, USA

## Research Objective

Medically underserved groups, such as the uninsured, racial and ethnic minorities, and people with multiple chronic conditions, experience barriers in accessing health care. However, 91% of people in the United States use cell phones and 81% of cell phone users send and receive text messages. Based on evidence that reminder/recall improves adherence to clinical encounters, three text-message based initiatives were implemented in an integrated urban safety net healthcare system, with the aims of improving access to care, improving health care utilization, and improving patient satisfaction.

## Methods

A software platform was used to automate sending and receiving text messages. Questions about text messaging were added to CAHPS patient satisfaction surveys, which were fielded by a certified vendor. Clinical and process outcomes were assessed through examining text message response rates and rates of seasonal flu immunization, well child check visits attended, and primary care appointments kept, cancelled, or for which patients did not show among program participants as compared to non-participants.

## Summary of Findings

Over 15,000 patients chose to enroll across the three programs, together receiving almost 125,000 text messages as of May 31, 2014. Patients enrolled in appointment reminders at 5 times the rate of the other two programs combined. Statistically significant improvements were observed in primary care visit outcome rates and in adherence to well child check guidelines. HIPAA Omnibus Rule and Telephone Consumer Protection Act legislative and regulatory changes substantially influenced consent processes, allowable message content, and operational practices. Variation in clinic practices were discovered during implementation, resulting in process refinements.

## D&I impact

Text messaging represents a potentially low-cost way to improve between-visit engagement and population health. The results of this project describe how a low-cost, high-access technology solution can be effectively implemented in a safety net setting that predominantly cares for underserved populations.

